# Lack of Association between Common *LAG3/CD4* Variants and Risk of Migraine

**DOI:** 10.3390/ijms24021292

**Published:** 2023-01-09

**Authors:** Elena García-Martín, Santiago Navarro-Muñoz, Pedro Ayuso, Christopher Rodríguez, Mercedes Serrador, Hortensia Alonso-Navarro, Marisol Calleja, Francisco Navacerrada, Laura Turpín-Fenoll, Marta Recio-Bermejo, Rafael García-Ruiz, Jorge Millán-Pascual, José Francisco Plaza-Nieto, Esteban García-Albea, José A. G. Agúndez, Félix Javier Jiménez-Jiménez

**Affiliations:** 1 University Institute of Molecular Pathology Biomarkers, ARADyAL. Instituto de Salud Carlos III, Universidad de Extremadura, E-10071 Cáceres, Spain; 2 Section of Neurology, Hospital La Mancha-Centro, E-13600 Alcázar de San Juan, Spain; 3 Department of Family Medicine, Hospital “Príncipe de Asturias”, Universidad de Alcalá, E-28801 Alcalá de Henares, Spain; 4 Section of Neurology, Hospital Universitario del Sureste, E-28500 Arganda del Rey, Spain; 5 Department of Medicine-Neurology, Universidad de Alcalá, E-28801 Alcalá de Henares, Spain

**Keywords:** migraine, inflammatory factors, genetics, genetic polymorphisms, *LAG3* gene, *CD4* gene, risk factors

## Abstract

Several papers have been published suggesting a probable role of inflammatory factors in the etiopathogenesis of migraine. In this study, we investigated the possible association between common variants in the *LAG3/CD4* genes (both genes, which are closely related, encode proteins involved in inflammatory and autoimmune responses) in the risk of migraine in a cohort of Caucasian Spanish participants. For this purpose, the frequencies of *CD4* rs1922452, *CD4* rs951818, and *LAG3* rs870849 genotypes and allelic variants, using a specific *TaqMan*-based qPCR assay, were assessed in 290 patients diagnosed with migraine and in 300 healthy controls. The relationship of these variables with several clinical features of migraine was also analyzed. The frequencies of the analyzed *LAG3/CD4* genotypes did not differ significantly between the two study groups and were not related to the sex, age at onset of migraine, family history of migraine, presence or absence of aura, or the triggering effect of ethanol on migraine episodes. These results suggest a lack of association between common variants in the *LAG3/CD4* genes and the risk of developing migraine in the Caucasian Spanish population.

## 1. Introduction

Migraine is one of the most frequent neurological disorders affecting 10–18% of the population (2–3 women for each man). Positive family history is very frequent, both in migraine with aura (MWA) and in migraine without aura (MWoA), accounting for 50–70% of cases, and subjects with first-degree parents affected with MWA or MWoA have increased risk for these conditions. Despite this fact, which suggests an important role of genetic factors in the risk of migraine, the genetics of this disease is not well established, except for the identification of *CACNA1A*, *ATP1A2*, and *SCN1A* genes as causative for familial hemiplegic migraine [1]. The most recent meta-analysis of hypothesis-free Genome-Wide Association Studies (GWAS), involving a total of 102,084 migraine cases and 771,257 controls, identified 123 susceptibility loci (86 of them were previously unknown) for migraine [2]. An important number of hypothesis-driven case–control association studies reported during the last 25 years, which involved many candidate genes, have shown inconsistent and variable results regarding association with migraine. A detailed revision of these studies is out of the scope of the present article. Despite there being many studies regarding the pathogenetic mechanisms of migraine, the pathophysiology of this disease has not been fully elucidated. Many recent reports, as described in the discussion, suggest an important role of inflammatory and immunity factors. 

LAG3 protein is encoded by the *lymphocyte activation gene 3* (*LAG3* or *CD223* gene; chromosome 12p13.31; gene ID 3902, MIM 153337). The main mechanism of action of LAG3 protein, which is expressed by regulatory T cells (both exhausted and activated CD4+ and CD8+ T cells) and microglia, is the delivering of inhibitory signals involved in the regulation of immune cell homeostasis, T cell proliferation and activation, cytokines production, cytolytic activity, and other functions related with inflammatory responses [3,4]. The CD4 membrane glycoprotein of T lymphocytes, encoded by the *CD4 molecule* gene (*CD4*; gene ID 920; MIM 186940), is an important mediator of immune and inflammatory responses as well. CD4 and LAG3 molecules are closely related, both at the gene and protein levels [5]. 

Despite LAG3 and CD4 not having been the matter of study in migraine patients, the possible role of inflammatory factors in migraine, together with the important role of these proteins in the inflammatory and immune response, suggest that LAG3 and CD4 could be interesting as markers of migraine. The main single nucleotide polymorphisms (SNVs) in *LAG3* and *CD4* genes have been related to protection against the severity of primary immune thrombocytopenia (*LAG3* rs870849 T>C) [6], comorbidity in multiple sclerosis (*CD4* rs1922452 A>G) [7], and disease progression and mortality of sepsis (*CD4* rs951818 C>A) [8]. Two recent case–control association studies in different populations have shown an association between *CD4* rs1922452 and *CD4* rs951818 and the risk of Parkinson’s disease [9,10]. 

The present study aims to establish whether these three common SNVs in the *LAG3* and *CD4* genes are associated with the risk for migraine.

## 2. Results

The frequencies of *CD4 rs1922452, CD4 rs951818, and LAG3 rs870849* genotypes and allelic variants, that were in Hardy–Weinberg’s equilibrium, both in migraine patients and in healthy controls groups, did not differ significantly between the two groups, both considering the whole group (Table 1), and when analyzing each sex separately (Supplementary Table S1). The genotype and allele frequencies in patients with migraine did not differ significantly when dividing into two subgroups according to the age at onset of migraine (≤15 vs. ≥16 years, Supplementary Table S2), positive vs. negative family history for migraine (Supplementary Table S2), presence vs. absence of aura (Supplementary Table S2), or the triggering effect of ethanol on migraine attacks (Supplementary Table S3). Finally, the mean ± SD age at the onset of migraine did not differ significantly between the three genotypes of each of the SNVs analyzed (Table 2). 

## 3. Discussion

The probable role of inflammatory factors in the pathogenesis of migraine has been suggested, at least, by the following data:

(1) Description of the possible involvement of cytokines in the proposed inflammatory mechanisms of migraine [11], suggested by the increased serum levels of pro-inflammatory cytokines (tumor necrosis factor α -TNF-α-, interleukin 6- IL-6-, and IL-1β), and transforming growth factor beta 1 (TGF1β), and decreased serum levels of the anti-inflammatory cytokine IL-10 [12,13], compared with controls. TNF-α is increased in the cerebrospinal fluid of patients with chronic migraine as well [14].
(2) Description of serum increased C reactive protein (CRP) concentrations in patients with migraine compared with controls, both in MWA and in MWoA [12,15,16].
(3) Description of increased serum brain-derived neurotrophic factor (BDNF) levels in migraine patients during migraine attacks compared to headache-free periods, tension-type headaches, and healthy controls [11,17], increased BDNF levels in the cerebrospinal fluid [17], increased levels of nerve growth factor (NGF) in the serum/plasma, saliva, and cerebrospinal fluid [17], and decreased BDNF and NGF in platelets from migraine patients compared with controls [17]. BDNF and NGF have a role in the modulation of inflammatory processes.
(4) Relation of endogenous neuropeptides with known anti-inflammatory effects, such as the calcitonin gene-related peptide (CGRP, present in perivascular trigeminal sensory afferents and the cerebral arterial walls of the circle of Willis), and pituitary adenylate cyclase-activating peptide-38 (PACAP-38, found in perivascular trigeminal nerve fibers and ganglia, the sphenopalatine ganglion, and the trigeminal *nucleus caudalis*) with the pathogenesis of migraine [18,19]. In addition, TNF-α seems to be a mediator of CGRP transcription [20].
(5) Relation of transient receptor potentials vanilloids (TRPV) with inflammatory mechanisms of migraine and several *TRPV* genes with the risk for migraine [11,17].
(6) Finding of microglial and parameningeal inflammatory activity in MWA, but not in MWoA, in neuroimaging studies [21].
(7) Clinical–epidemiological, biochemical, experimental, and pharmacological data give support to the hypothesis of a possible contribution of histamine and mast cells in the etiopathogenesis or the clinical presentation of migraine [22,23,24,25,26,27].
(8) Description of alterations in some microRNAs (miRNAs, important regulators to control inflammation), mainly miR-590-5p in migraine patients and animal models of migraine [28].
(9) Description of changes in serum levels of neuronal-specific enolase (NSE) and S100 calcium-binding protein B (S100B) in patients with migraine, although the changes found were inconsistent [11].
(10) Description of the important role of Toll-like receptors (TLRs), which play a significant role in immune and inflammatory responses, and are expressed by microglia and astrocytes, in the migraine etiology by activation of the microglia [29].

For this reason, the investigation of possible genetic susceptibility factors for migraine related to inflammation seems reasonable. In this regard, the proteins encoded by *LAG3/CD4* genes play an important role in inflammatory and immune responses. Moreover, at least one SNV in these genes has been related to the risk of a paradigm of inflammatory and neurodegenerative diseases, such as multiple sclerosis [7].

The results of the current study, which involved Caucasian Spanish people, did not show any major association of common SNVs *CD4* rs1922452, *CD4* rs951818, or *LAG3* rs870849 with the risk of migraine. In addition, none of the SNVs studied were related to sex, age at onset of migraine attacks, family history of migraine, presence or absence of aura, or triggering of migraine attacks by ethanol. 

The current study, however, has as its main limitation the relatively low sample size, both of the group of patients with migraine and the group of healthy controls. While the sample size would be sufficiently powered to detect ORs equal to or greater than 1.5, it might not be sufficient to detect more modest associations. However, and taking into account this main limitation, this study suggests the absence of association between *CD4* rs1922452, *CD4* rs951818, and *LAG3* rs870849 SNVs and the risk of developing migraine in the Spanish Caucasian population.

## 4. Patients and Methods

### 4.1. Patients and Controls

We studied the genotype and allelic variants *CD4* rs1922452, *CD4* rs951818, and *LAG3* rs870849 in 290 patients fulfilling standardized diagnostic criteria for migraine [30], and 300 age- and sex-matched controls. Patients diagnosed with migraine (not suffering from other headache types or other neurological diseases) were recruited from the general neurologic clinics of several University Hospitals during 2 periods: 197 patients that were recruited between September 2006–September 2007 and 93 between June 2017 and February 2019. Healthy controls (included in the study if they had neither personal nor family history of migraine and they did not suffer from another type of headaches), most of them staff or students from the University of Extremadura, were recruited during the same periods (215 in the first and 85 in the second ones). 

### 4.2. Ethical Aspects

This study was conducted according to the principles of the Declaration of Helsinki and was approved by the Ethics Committees of the Hospital La Mancha-Centro (Alcázar de San Juan, Ciudad Real, Spain, Ref 03/2016), University Hospital “Príncipe de Asturias” (2007 no reference number, and LIB 04/2015; Alcalá de Henares, Madrid, Spain), University Hospital of Badajoz (2007, no reference number; Badajoz, Spain), and the Ethics Committee of the province of Cáceres (2016, no reference number, Cáceres, Spain). Participants signed informed consent (after explaining the objectives and procedure) before their inclusion in the study.

### 4.3. Genotyping of CD4 rs1922452, CD4 rs951818, and LAG3 rs870849 Variants

Genomic DNA, obtained from peripheral leukocytes of venous blood samples from migraine patients and controls, was used to perform genotyping studies. Analysis was performed by using real-time PCR (Applied Biosystems 7500 qPCR thermocycler, Applied Biosciences Hispania, Alcobendas, Madrid, Spain) with specific custom-designed TaqMan probes (Life Technologies, Alcobendas, Madrid, Spain). We analyzed the same single nucleotide variations (SNVs) studied in other case–control studies involving patients with other neurological diseases [7,9,10]; that is, one intronic and one non-coding transcript exonic SNV with high allele frequencies, and the only missense SNVs with an allele frequency over 0.01 in the population analyzed here. The SNVs were analyzed, and the corresponding TaqMan tests performed were, respectively, rs1922452 (C__11914936_10), rs951818 (C___8921385_10), and rs870849 (C___9797874_10).

### 4.4. Statistical Analysis

The SPSS 27.0 version for Windows (SPSS Inc., Chicago, IL, USA) was used to perform the statistical analysis, and the online program https://ihg.gsf.de/cgi-bin/hw/hwa1.pl (last accessed, 1 November 2022) to confirm the Hardy–Weinberg equilibrium, both in patients diagnosed with migraine and in controls. The intergroup comparison values were calculated by using the chi-square test, or Fisher’s exact test where appropriate. We calculated the 95% confidence intervals, and the negative predictive values as well [31]. The false discovery rate (FDR) was used to perform the correction for multiple comparison adjustments [32].

Calculation of the sample size was performed by using a genetic model analyzing the frequency of the lower allele with an odds ratio (OR) value = 1.5 (α = 0.05) from the allelic frequencies found in healthy subjects. According to the sample size of this study, the statistical power (two-tailed association) for variant alleles was 91.3% for rs1922452, 93.3% for rs951818, and 92.9% for rs870849.

We also performed a secondary analysis on the possible influence of the frequency of the genotype or allelic variants of migraine patients according to the age at onset of migraine attacks (≤15 years vs. ≥16 years), positivity vs. negativity of family history of migraine, presence vs. absence of aura, and triggering vs. not triggering of migraine attacks with alcohol (Table 3). We used the chi-square test, Fisher’s exact test, or the student t-test when appropriate.

## Figures and Tables

**Table 1:** Genotypes and allelic variants of patients with migraine and healthy volunteers. The values in each cell represent the number (percentage; 95% confidence intervals). P: crude probability; Pc: probability after multiple comparisons; NPV: negative predictive value.

GENOTYPE	PATIENTS (N = 290, 580 Alleles)	CONTROLS (N = 300, 600 Alleles)	OR (95% CI), P; Pc; NPV (95% CI)
rs1922452 A/A	57 (19.7; 15.1–24.2)	48 (16.0; 11.9–20.1)	1.28 (0.84–1.96); 0.246; 0.859; 0.52 (0.50–0.54)
rs1922452 A/G	131 (45.2; 39.4–50.9)	144 (48.0; 42.3–53.7)	0.89 (0.65–1.23); 0.492; 0.859; 0.50 (0.46–0.53)
rs1922452 G/G	102 (35.2; 29.7–40.7)	108 (36.0; 30.6–41.4)	0.97 (0.69–1.35); 0.834; 0.859; 0.51 (0.47–0.54)
rs951818 A/A	95 (32.8; 27.4–38.2)	104 (34.7; 29.3–40.1)	0.92 (0.65–1.29); 0.624; 0.859; 0.50 (0.47–0.53)
rs951818 A/C	140 (48.3; 42.5–54.0)	148 (49.3; 43.7–55.0)	0.96 (0.69–1.32; 0.797; 0.859; 0.50 (0.46–0.54)
rs951818 C/C	55 (19.0; 14.5–23.5)	48 (16.0; 11.9–20.1)	1.23 (0.80–1.88); 0.343; 0.859; 0.52 (0.50–0.54)
rs870849 C/C	112 (38.6; 33.0–44.2)	118 (39.3; 33.8–44.9)	0.97 (0.70–1.35); 0.859; 0.859; 0.51 (0.47–0.54)
rs870849 C/T	140 (48.3; 42.5–54.0)	138 (46.0; 40.4–51.6)	1.10 (0.79–1.51); 0.580; 0.859; 0.52 (0.48–0.56)
rs870849 T/T	38 (13.1; 9.2–17.0)	44 (14.7; 10.7–18.7)	0.88 (0.55–1.40); 0.584; 0.859; 0.50 (0.49–0.52)
**ALLELES**			
rs1922452 A	245 (42.2; 38.2–46.3)	240 (40.0; 36.1–43.9)	1.10 (0.87–1.38); 0.434; 0.651; 0.52 (0.49–0.54)
rs1922452 G	335 (57.8; 53.7–61.8)	360 (60.0; 56.1–63.9)	0.91 (0.72–1.15); 0.434; 0.651; 0.50 (0.46–0.53)
rs951818 A	330 (56.9; 52.9–60.9)	356 (59.3; 55.4–63.3)	0.91 (0.72–1.14); 0.397; 0.651; 0.49 (0.46–0.53)
rs951818 C	250 (43.1; 39.1–47.1)	244 (40.7; 36.7–44.6)	1.11 (0.88–1.39); 0.397; 0.651; 0.52 (0.49–0.54)
rs870849 C	364 (62.8; 58.8–66.7)	374 (62.3; 58.5–66.2)	1.11 (0.80–1.29); 0.880; 0.880; 0.51 (0.47–0.55)
rs870849 T	216 (37.2; 33.3–41.2)	226 (37.7; 33.8–41.5)	0.98 (0.78–1.24); 0.880; 0.880; 0.51 (0.48–0.53)

**Table 2:** Mean (SD) age at onset of migraine for the different genotypes.

	AGE AT ONSET (SD); Range	*t*-Test *p*-Value	*t*-Test *p*-Value
**GENOTYPES**		rs1922452 A/G	rs1922452 G/G
rs1922452 A/A	17.28 (10.91); 5–67	0.701	0.699
rs1922452 A/G	17.95 (11.09); 2–56		0.996
rs1922452 G/G	17.96 (10.43); 4–52		
**GENOTYPES**		rs951818 A/C	rs951818 C/C
rs951818 A/A	18.08 (11.35); 4–67	0.806	0.806
rs951818 A/C	17.72 (10.90); 4–56		0.960
rs951818 C/C	17.64 (9.65); 2–48		
**GENOTYPES**		rs870849 C/T	rs870849 T/T
rs870849 C/C	18.49 (12.13); 4–67	0.255	0.764
rs870849 C/T	16.93 (9.59); 2–49		0.218
rs870849 T/T	19.16 (10.77); 4–50		

**Table 3:** Demographic and clinical data of the series studied.

Group	Migraine Patients (n = 290)	Healthy Controls (n = 300)
**Age (years): mean (SD); range**	38.8 (13.7); 13–73	38.9 (13.4); 19–77
**Age at onset (years): mean (SD); range**	17.8 (11.0); 2–67	NA
**Age at onset <15 years: N (%)**	155 (53.4%)	NA
**Female N (%)**	211 (72.8)	218 (72.7)
**Positive family history: N (%)**	220 (75.9)	NA
**Aura: N (%)**	144 (49.6)	NA

## Data Availability

All data relating to the current study, intended for reasonable use, are available from J.A.G. Agúndez (University Institute of Molecular Pathology Biomarkers, University of Extremadura -UNEx ARADyAL Instituto de Salud Carlos III, Av/ de la Universidad S/N, E10071 Cáceres. Spain), and F.J. Jiménez-Jiménez (Section of Neurology, Hospital del Sureste, Arganda del Rey, Madrid, Spain).

## Supplementary Materials

The following supporting information can be downloaded at: https://www.mdpi.com/article/10.3390/ijms24021292/s1.
