# Supplementary material for: Lack of Association between Common LAG3/CD4 Variants and Risk of Migraine
Source: Int J Mol Sci. 2023 Jan 9;24(2):1292. doi: 10.3390/ijms24021292 (PMC9866744; doi:10.3390/ijms24021292)
Supplement: Supplementary file 1 [file ijms-24-01292-s001.zip › ijms-2075841-supplementary 1.0.pdf]

International Journal of
Molecular Sciences

MDPI

Supplementary Table 1. Genotypes and allelic variants of patients with migraine and healthy volunteers distributed by sex. The values in each cell represent the number (percentage; 95% confidence intervals).

GENOTYPE	*MIGRAINE WOMEN (N=211, 422 ALLELES)*	*CONTROL WOMEN (N=218, 436 ALLELES)*	*INTERGROUP COMPARISON VALUES OR (95%CI) P*	*NPV*	*MIGRAINE MEN (N=79, 158 ALLELES)*	*CONTROL MEN (N=82, 164 ALLELES)*	*INTERGROUP COMPARISON VALUES OR (95%CI) P*	*NPV*
rs1922452 A/A	43 (20.4; 14.9-25.8)	35 (16.1; 11.2-20.9)	1.34 (0.82-2.20); 0.246	0.52 (0.50-0.54)	14 (17.7; 9.3-26.1)	13 (15.9; 7.9-23.8)	1.14 (0.50-2.62); 0.752	0.52 (0.48-0.55)
rs1922452 A/G	90 (42.7; 36.0-49.3)	107 (49.1; 42.4-55.7)	0.77 (0.53-1.13); 0.182	0.48 (0.43-0.52)	41 (51.9; 40.9-62.9)	37 (45.1; 34.4-55.9)	1.31 (0.71-2.44); 0.391	0.54 (0.46-0.62)
rs1922452 G/G	78 (37.0; 30.5-43.5)	76 (34.9; 28.5-41.2)	1.10 (0.74-1.63); 0.650	0.52 (0.48-0.55)	24 (30.4; 20.2-40.5)	32 (39.0; 28.5-49.6)	0.69 (0.36-1.31); 0.251	0.48 (0.42-0.54)
rs951818 A/A	69 (32.7; 26.4-39.0)	76 (34.9; 28.5-41.2)	0.91 (0.61-1.34); 0.637	0.50 (0.47-0.54)	26 (32.9; 22.5-43.3)	28 (34.1; 23.9-44.4)	0.95 (0.49-1.82); 0.869	0.51 (0.45-0.56)
rs951818 A/C	101 (47.9; 41.1-54.6)	107 (49.1; 42.4-55.7)	1.01 (0.69-1.47); 0.962	0.50 (0.45-0.55)	39 (49.4; 38.3-60.4)	41 (50.0; 39.2-60.8)	0.98 (0.53-1.81); 0.936	0.51 (0.42-0.59)
rs951818 C/C	41 (19.4; 14.1-24.8)	35 (16.1; 11.2-20.9)	1.27 (0.77-2.07); 0.360	0.52 (0.50-0.54)	14 (17.7; 9.3-26.1)	13 (15.9; 7.9-23.8)	1.14 (0.50-2.62); 0.752	0.52 (0.48-0.55)
rs870849 C/C	86 (40.8; 34.1-47.4)	84 (38.5; 32.1-45.0)	1.10 (0.75-1.62); 0.638	0.52 (0.48-0.56)	26 (32.9; 22.5-43.3)	34 (41.5; 30.8-52.1)	0.69 (0.36-1.32); 0.263	0.48 (0.41-0.54)
rs870849 C/T	98 (46.4; 39.7-53.2)	101 (46.3; 39.7-52.9)	1.01 (0.69-1.47); 0.981	0.51 (0.46-0.56)	42 (53.2; 42.2-64.2)	37 (45.1; 34.4-55.9)	1.38 (0.74-2.57); 0.309	0.55 (0.47-0.63)
rs870849 T/T	27 (12.8; 8.3-17.3)	33 (15.1; 10.4-19.9)	0.82 (0.48-1.42); 0.485	0.50 (0.48-0.52)	11 (13.9; 6.3-21.6)	11 (13.4; 6.0-20.8)	1.04 (0.43-2.57); 0.925	0.51 (0.48-0.54)
**ALLELE**								
rs1922452 A	176 (41.7; 37.0-46.4)	177 (40.6; 36.0-45.2)	1.05 (0.80-1.37); 0.741	0.51 (0.48-0.54)	69 (43.7; 35.9-51.4)	63 (38.4; 31.0-45.9)	1.24 (0.80-1.94); 0.338	0.53 (0.48-0.58)
rs1922452 G	246 (58.3; 53.6-63.0)	259 (59.4; 54.8-64.0)	0.96 (0.73-1.25); 0.741	0.50 (0.46-0.54)	89 (56.3; 48.6-64.1)	101 (61.6; 54.1-69.0)	0.81 (0.52-1.26); 0.338	0.48 (0.41-0.55)
rs951818 A	239 (56.6; 51.9-61.4)	259 (59.4; 54.8-64.0)	0.89 (0.68-1.17); 0.412	0.49 (0.45-0.53)	91 (57.6; 49.9-65.3)	97 (59.1; 51.6-66.7)	0.94 (0.60-1.46); 0.778	0.50 (0.43-0.57)
rs951818 C	183 (43.4; 38.6-48.1)	177 (40.6; 36.0-45.2)	1.12 (0.85-1.47); 0.412	0.52 (0.49-0.55)	67 (42.4; 34.7-50.1)	67 (40.9; 33.3-48.4)	1.07 (0.68-1.66); 0.778	0.52 (0.47-0.56)
rs870849 C	270 (64.0; 59.4-68.6)	269 (61.7; 57.1-66.3)	1.10 (0.84-1.46); 0.489	0.52 (0.48-0.57)	94 (59.5; 51.8-67.1)	105 (64.0; 56.7-71.4)	0.83 (0.53-1.29); 0.404	0.48 (0.41-0.55)
rs870849 T	152 (36.0; 31.4-40.6)	167 (38.3; 33.7-42.9)	0.91 (0.69-1.20);0.489	0.50 (0.47-0.53)	64 (40.5; 32.9-48.2)	59 (36.0; 28.6-43.3)	1.21 (0.77-1.90); 0.404	0.53 (0.48-0.57)

Suplementary Table 2. Genotypes and allelic variants of patients with migraine and healthy volunteers distributed by age of onset, the positivity of family history of migraine, and presence or absence of aura. The values in each cell represent the number (percentage; 95% confidence intervals).

GENOTYPE	AGE AT ONSET ≤ 15 YEARS (N= 155, 310 alleles)	AGE AT ONSET ≥ 16 YEARS (N=135, 270 alleles)	*INTERGROUP COMPARISON VALUES OR (95%CI); P; NPV*	POSITIVE FAMILY HISTORY OF MIGRAINE (N=220, 440 alleles)	NEGATIVE FAMILY HISTORY OF MIGRAINE (N=66, 132 alleles)	*INTERGROUP COMPARISON VALUES OR (95%CI); P; NPV*	MIGRAINE WITH AURA (N=144, 288 alleles)	MIGRAINE WITHOUT AURA (N=142, 284 alleles)	*INTERGROUP COMPARISON VALUES OR (95%CI); P ; NPV*
rs1922452 A/A	31 (20.0; 13.7-26.3)	26 (19.3; 12.6-25.9)	1.05 (0.59-1.87); 0.874; 0.47 (0.44-0.50)	43 (19.5; 14.3-24.8)	13 (19.7; 10.1-29.3)	0.99 (0.50-1.98); 0.978; 0.23 (0.20-0.25)	25 (17.4; 11.2-23.5)	32 (22.5; 15.7-29.4)	0.72 (0.40-1.30); 0.274; 0.48 (0.45-0.51)
rs1922452 A/G	72 (46.5; 38.6-54.3)	59 (43.7; 35.3-52.1)	1.12 (0.70-1.78); 0.640; 0.48 (0.42-0.53)	95 (43.2; 36.6-49.7)	35 (53.0; 41.0-65.1)	0.67 (0.39-1.17); 0.159; 0.20 (0.15-0.25)	70 (48.6; 40.4-56.8)	58 (40.8; 32.8-48.9)	1.37 (0.86-2.19); 0.187; 0.53 (0.48-0.59)
rs1922452 G/G	52 (33.5; 26.1-41.0)	50 (37.0; 28.9-45.2)	0.86 (0.53-1.39); 0.536; 0.45 (0.41-0.50)	82 (37.3; 30.9-43.7)	18 (27.3; 16.5-38.0)	1.59 (0.86-2.91); 0.136; 0.26 (0.22-0.29)	49 (34.0; 26.3-41.8)	52 (36.6; 28.7-44.5)	0.89 (0.55-1.45); 0.647; 0.49 (0.44-0.53)
rs951818 A/A	47 (30.3; 23.1-37.6)	48 (35.6; 27.5-43.6)	0.79 (0.48-1.29) ; 0.344 ; 0.45 (0.40-0.49)	68 (30.9; 24.8-37.0)	23 (34.8; 23.4-46.3)	0.84 (0.47-1.50); 0.547; 0.22 (0.18-0.26)	46 (31.9; 24.3-39.6)	49 (34.5; 26.7-42.3)	0.89 (0.54-1.46); 0.646; 0.49 (0.40-0.57)
rs951818 A/C	80 (51.6; 43.7-59.5)	60 (44.4; 36.1-52.8)	1.33 (0.84-2.12); 0.224; 0.50 (0.44-0.56)	111 (50.5; 43.8-57.1)	29 (43.9; 32.0-55.9)	1.30 (0.75-2.26); 0.354; 0.285 (0.20-0.30)	70 (48.6; 40.4-56.8)	68 (47.9; 39.7-56.1)	1.03 (0.65-1.64); 0.903; 0.50 (0.44-0.56)
rs951818 C/C	28 (18.1; 12.0-24.1)	27 (20.0; 13.3-26.7)	0.88 (0.49-1.59); 0.675; 0.46 (0.43-0.49)	41 (18.6; 13.5-23.8)	14 (21.2; 11.3-31.1)	0.85 (0.43-1.68); 0.642; 0.23 (0.20-0.25)	28 (19.4; 13.0-25.9)	25 (17.6; 11.3-23.9)	1.13 (0.62-2.05); 0.690; 0.50 (0.47-0.53)
rs870849 C/C	59 (38.1; 30.4-45.7)	53 (39.3; 31.0-47.5)	0.95 (0.59-1.53); 0.835; 0.46 (0.41-0.51)	84 (38.2; 31.8-44.6)	26 (39.4; 27.6-51.2)	0.95 (0.54-1.67); 0.859; 0.23 (0.19-0.27)	61 (42.4; 34.3-50.4)	49 (34.5; 26.7-42.3)	1.40 (0.86-2.25); 0.173; 0.53 (0.48-0.58)
rs870849 C/T	78 (50.3; 42.5-58.2)	62 (45.9; 37.5-54.3)	1.19 (0.75-1.89); 0.456; 0.49 (0.43-0.55)	107 (48.6; 42.0-55.2)	31 (47.0; 34.9-59.0)	1.07 (0.62-1.86); 0.812; 0.24 (0.19-0.29)	67 (46.5; 38.4-54.7)	71 (50.0; 41.8-58.2)	0.87 (0.55-1.38); 0.558; 0.48 (0.42-0.54)
rs870849 T/T	18 (11.6; 6.6-16.7)	20 (14.8; 8.8-20.8)	0.76 (0.38-1.50); 0.421; 0.46 (0.43-0.48)	29 (13.2; 8.7-17.7)	9 (13.6; 5.4-21.9)	0.96 (0.43-2.15); 0.924; 0.23 (0.21-0.25)	16 (11.1; 6.0-16.2)	22 (15.5; 9.5-21.4)	0.68 (0.34-1.36); 0.276; 0.48 (0.46-0.51)
ALLELES									
rs1922452 A	134 (43.2; 37.7-48.7)	111 (41.1; 35.2-47.0)	1.09 (0.78-1.52); 0.607; 0.48 (0.44-0.51)	181 (41.1; 36.5-45.7)	61 (46.2; 37.7-54.7)	0.81 (0.55-1.20); 0.301; 0.22 (0.18-0.25)	120 (41.7; 36.0-47.4)	122 (43.0; 37.2-48.7)	0.95 (0.68-1.32); 0.755; 0.49 (0.45-0.53)
rs1922452 G	176 (56.8; 51.3-62.3)	159 (58.9; 53.0-64.8)	0.92 (0.66-1.28); 0.607; 0.45 (0.40-0.50)	259 (58.9; 54.3-63.5)	71 (53.8; 45.3-62.3)	1.23 (0.83-1.82); 0.301; 0.25 (0.21-0.30)	168 (58.3; 52.6-64.0)	162 (57.0; 51.3-62.8)	1.05 (0.76-1.47); 0.755; 0.50 (0.45-0.55)
rs951818 A	174 (56.1; 50.6-61.7)	156 (57.8; 51.9-63.7)	0.94 (0.67-1.30); 0.689; 0.46 (0.41-0.51)	247 (56.1; 51.5-60.8)	75 (56.8; 48.4-65.3)	0.97 (0.66-1.44); 0.890; 0.23 (0.19-0.27)	162 (56.3; 50.5-62.0)	166 (58.5; 52.7-64.2)	0.91 (0.66-1.27); 0.595; 0.48 (0.43-0.53)

rs951818 C	136 (43.9; 38.3-49.4)	114 (42.2; 36.3-48.1)	1.07 (0.77-1.49); 0.689; 0.47 (0.44-0.51)	193 (43.9; 39.2-48.5)	57 (43.2; 34.7-51.6)	1.03 (0.69-1.52); 0.890; 0.23 (0.20-0.26)	126 (43.8; 38.0-49.5)	118 (41.5; 35.8-47.3)	1.09 (0.79-1.52); 0.595; 0.51 (0.47-0.54)
rs870849 C	196 (63.2; 57.9-68.6)	168 (62.2; 56.4-68.0)	1.04 (0.75-1.46); 0.803; 0.47 (0.42-0.53)	275 (62.5; 58.0-67.0)	83 (62.9; 54.6-71.1)	0.98 (0.66-1.47); 0.937; 0.23 (0.18-0.28)	189 (65.6; 60.1-71.1)	169 (59.5; 53.8-65.2)	1.30 (0.93-1.82); 0.131; 0.54 (0.48-0.59)
rs870849 T	114 (36.8; 31.4-42.1)	102 (37.8; 32.0-43.6)	0.96 (0.68-1.34); 0.803; 0.46 (0.43-0.49)	165 (37.5; 33.0-42.0)	49 (37.1; 28.9-45.4)	1.02 (0.68-1.52); 0.937; 0.23 (0.20-0.26)	99 (34.4; 28.9-39.9)	115 (40.5; 34.8-46.2)	0.77 (0.55-1.80); 0.131; 0.47 (0.44-0.51)

Supplementary Table 3. Genotypes and allelic variants of patients with migraine and their relationship with response to ethanol as a triggering factor. The values in each cell represent the number (percentage; 95% confidence intervals).

GENOTYPE	Triggering effect of ethanol (92 individuals, 184 alleles)	Lack of effect of ethanol (198 individuals, 396 alleles)	*INTERGROUP COMPARISON VALUES OR (95%CI); P; NPV*
rs1922452 A/A	18 (19.6; 11.5-27.7)	39 (19.7; 14.2-25.2)	0.99 (0.53-1.85); 0.979; 0.68 (0.66-0.71)
rs1922452 A/G	39 (42.4; 32.3-52.5)	92 (46.5; 39.5-53.4)	0.85 (0.52-1.40); 0.517; 0.67 (0.62-0.72)
rs1922452 G/G	35 (38.0; 28.1-48.0)	67 (33.8; 27.2-40.4)	1.20 (0.72-2.01); 0.486; 0.70 (0.66-0.74)
rs951818 A/A	24 (26.1; 17.1-35.1)	71 (35.9; 29.2-42.5)	0.63 (0.37-1.09); 0.100; 0.65 (0.62-0.69)
rs951818 A/C	49 (53.3; 43.1-63.5)	91 (46.0; 39.0-52.9)	1.34 (0.82-2.20); 0.248; 0.71 (0.66-0.77)
rs951818 C/C	19 (20.7; 12.4-28.9)	36 (18.2; 12.8-23.6)	1.17 (0.63-2.18); 0.618; 0.69 (0.66-0.72)
rs870849 C/C	32 (34.8; 25.1-44.5)	80 (40.4; 33.6-47.2)	0.79 (0.47-1.32); 0.361; 0.66 (0.62-0.71)
rs870849 C/T	48 (52.2; 42.0-62.4)	92 (46.5; 39.5-53.4)	1.26 (0.77-2.06); 0.366; 0.71 (0.65-0.76)
rs870849 T/T	12 (13.0; 6.2-19.9)	26 (13.1; 8.4-17.8)	0.99 (0.48-2.07); 0.984; 0.68 (0.66-0.71)
ALLELES			
rs1922452 A	75 (40.8; 33.7-47.9)	170 (42.9; 38.1-47.8)	0.92 (0.64-1.31); 0.623; 0.68 (0.64-0.71)
rs1922452 G	109 (59.2; 52.1-66.3)	226 (57.1; 52.2-61.9)	1.09 (0.77-1.56); 0.623; 0.69 (0.65-0.74)
rs951818 A	97 (52.7; 45.5-59.9)	233 (58.8; 54.0-63.7)	0.78 (0.55-1.11); 0.166; 0.65 (0.61-0.70)
rs951818 C	87 (47.3; 40.1-54.5)	163 (41.2; 36.3-46.0)	1.28 (0.90-1.82); 0.166; 0.71 (0.67-0.74)
rs870849 C	112 (60.9; 53.8-67.9)	252 (63.6; 58.9-68.4)	0.89 (0.62-1.27); 0.522; 0.67 (0.62-0.72)
rs870849 T	72 (39.1; 32.1-46.2)	144 (36.4; 31.6-41.1)	1.13 (0.79-1.61); 0.522; 0.69 (0.87-0.93)
